# Infection prevention and control strategies for multidrug-resistant Gram-negative bacteria in healthcare: a narrative review

**DOI:** 10.1093/jacamr/dlag176

**Published:** 2026-08-19

**Authors:** Gregory Weston, Priya Nori, Harjot K Singh, Krystina L Woods, Rachel Lee, Emily Frech Preciado, Justin Halim, Emine Alp Mese, David J Weber

**Affiliations:** Division of Infectious Diseases, Department of Medicine, Montefiore Medical Center, Albert Einstein College of Medicine, Bronx, NY 10467, USA; Division of Infectious Diseases, Department of Medicine, Montefiore Medical Center, Albert Einstein College of Medicine, Bronx, NY 10467, USA; Division of Infectious Diseases, Weill Cornell Medicine, New York, NY, USA; Department of Infection Prevention, Mount Sinai West, New York, NY, USA; Division of Infectious Diseases, Department of Medicine, Icahn School of Medicine, Mount Sinai, New York, NY, USA; Memorial Sloan Kettering Cancer Center, Weill Cornell Medical College, New York, NY, USA; Division of Infectious Diseases, Weill Cornell Medicine, New York, NY, USA; Department of Medicine, Montefiore Medical Center, Bronx, NY, USA; Department of Infectious Diseases and Clinical Microbiology, Faculty of Medicine, Ankara Yıldırım Beyazıt University, Ankara, Türkiye; Department of Infectious Diseases and Clinical Microbiology, Bilkent City Hospital, Ankara, Türkiye; Division of Infectious Diseases, University of North Carolina School of Medicine, Chapel Hill, NC, USA; Infection Prevention, University of North Carolina Hospitals, Chapel Hill, NC, USA

## Abstract

Antimicrobial-resistant Gram-negative bacilli (AMR GNB) are associated with poor patient outcomes, are increasing in prevalence and are recognized by national and international public health authorities as a major threat to human health. The most common resistance elements vary by organism, but resistant Enterobacterales, *Pseudomonas* and *Acinetobacter* species have reservoirs in the human colon, natural environment and healthcare settings. Persistence of these organisms on inanimate surfaces contributes to the risk of bacterial transmission in healthcare settings. Standard infection prevention practices including hand hygiene, low-level disinfection and use of isolation precautions are effective in interrupting transmission of AMR GNB. However, the optimal use of isolation precautions is an area of active reconsideration and research. High-risk equipment, such as duodenoscopes, can be difficult to disinfect and has been associated with hospital transmission. The immunocompromised and critical care populations are at higher risk of infection and may benefit from additional infection control interventions. Innovative approaches to reduce healthcare transmission have included screening surveillance with the use of isolation or decolonization protocols, but these approaches require further study. Antimicrobial stewardship remains a key strategy to reduce the pressure to select for AMR GNB in healthcare settings and the general environment. Low-resource settings can implement existing World Health Organization infection prevention tools to reduce transmission of AMR GNB infections. Although implementation of existing infection prevention and antimicrobial stewardship strategies can limit transmission of AMR GNB infections, development of new approaches is needed to curb increasing prevalence of AMR GNB.

## Introduction

Bacterial antimicrobial resistance (AMR) is a critical global public health challenge, associated with an estimated 4.71 million deaths worldwide in 2021.^1^ Multidrug-resistant Gram-negative bacteria (MDR-GNB), including Enterobacterales, *Pseudomonas aeruginosa* and *Acinetobacter baumannii*, are associated with high morbidity, mortality, prolonged hospitalizations and increased healthcare costs.^2–4^ These pathogens readily acquire and disseminate resistance determinants via mobile genetic elements, enabling rapid spread.^5^ Resistance patterns and underlying mechanisms vary geographically, underscoring the need for robust institutional and national surveillance to guide control strategies. Examples of the expansive spread of AMR pathogens include macrolide-resistant *Bordetella pertussis* in China and New Delhi metallo-β-lactamase (MBL)-producing Enterobacterales in New York City.^6,7^ Accordingly, the US CDC has designated carbapenem-resistant Enterobacterales (CRE) and *A. baumannii* (CRAB) as urgent threats, and MDR *P. aeruginosa* as a serious threat.^8^ Effective infection prevention and control programmes remain crucial to limiting transmission and mitigating the clinical and economic impact of AMR. This review highlights foundational and emerging infection prevention and control measures to reduce healthcare-associated transmission of MDR-GNB infections, with a focus on the acute care setting.

## Resistance mechanisms

Multidrug-resistant Enterobacterales, *P. aeruginosa* and *A. baumannii* each pose major infection control challenges due to diverse and evolving resistance mechanisms and increasing prevalence.^9^ Data from surveillance programmes estimate that MDR-GNB resistance to carbapenems has increased more rapidly than resistance to any other Gram-negative antibiotic class and was associated with 1.03 million deaths globally in 2021.^1^

Table 1 lists the predominant resistance mechanisms and preferred treatments among Enterobacterales, *P. aeruginosa* and *A. baumannii* according to the Infectious Diseases Society of America Guidance.^10^

**Table 1.** Antimicrobial resistant Gram-negative categories and resistance elements

Organism/IDSA category	Resistance mechanism	Key enzymes/Features	IDSA terminology	Preferred treatment per IDSA guidance
Enterobacterales (*E. coli*, *Klebsiella* spp.)	Extended-spectrum β-lactamases (ESBLs)	CTX-M (most common), SHV, TEM	ESBL-E	Carbapenems (preferred) for serious infections (e.g. meropenem); avoid ceftriaxone even if susceptible
	Plasmid-mediated AmpC	CMY, DHA	AmpC-producing Enterobacterales	Cefepime (if susceptible, low inoculum); carbapenems for severe infections
	Chromosomal AmpC (inducible)	*Enterobacter cloacae* complex, *Klebsiella aerogenes*, *Citrobacter freundii*	‘Moderate–high risk AmpC producers’	Avoid ceftriaxone; use cefepime or carbapenem depending on severity
	Serine carbapenemases	KPC, OXA-48-like	CRE	Ceftazidime-avibactam, meropenem-vaborbactam or imipenem-relebactam (if susceptible)
	Metallo-β-lactamases (MBLs)	NDM, VIM, IMP	MBL-producing CRE	Cefiderocol or ceftazidime-avibactam + aztreonam; NDM is rising in the USA
*Pseudomonas aeruginosa*	Efflux pump upregulation	Mex systems	Difficult-to-treat resistance (DTR-*P. aeruginosa*)	Use newer β-lactams if susceptible: ceftolozane-tazobactam, ceftazidime-avibactam, imipenem-relebactam
	Porin loss (OprD)	↓ carbapenem uptake	Carbapenem-resistant *P. aeruginosa*	Avoid imipenem if OprD loss; use alternative active agents
	AmpC overexpression (PDC)	Inducible/derepressed	Intrinsic resistance	Cefepime or newer β-lactams depending on susceptibility
	Acquired β-lactamases	ESBLs, carbapenemases (rare MBLs)	MDR/XDR *P. aeruginosa*	Tailor to susceptibilities; consider combination therapy in severe disease
*Acinetobacter baumannii*	OXA-type carbapenemases	OXA-23 (most common)	CRAB (carbapenem-resistant *A. baumannii*)	Sulbactam-containing regimens preferred (e.g. sulbactam-durlobactam if available; high-dose ampicillin-sulbactam)
	Aminoglycoside-modifying enzymes	AAC, ANT, APH	MDR phenotype	Limits aminoglycoside use
	Porin changes and efflux	↓ permeability	—	Contributes to extreme resistance
	Target-site mutations	gyrA/parC	—	Fluoroquinolone resistance
	Emerging MBLs	NDM, others	—	May require cefiderocol or combination therapy

AAC, aminoglycoside 6′-N-acetyltransferase; AmpC, ampicillinase; ANT, aminoglycoside nucleotidyltransferase; APH, aminoglycoside phosphotransferase; CMY, cephamycinase; CTX-M, cefotaximase; DHA, Dharan; gyrA, gene for A subunit of DNA gyrase; IMP, imipenemase; KPC, *Klebsiella pneumoniae* carbapenemase; MEX, multiple efflux; NDM, New Delhi metallo-β-lactamase; OprD, outer membrane protein D; OXA, oxacillinase; parC, gene for A subunit of DNA topoisomerase IV; PDC, *Pseudomonas*-derived cephalosporinase; SHV, sulfhydryl-variable; TEM, Temoneira; VIM, Verona integron encoded metallo-β-lactamase.

Enterobacterales, such as *Escherichia coli* and *Klebsiella* spp., acquire extended-spectrum β-lactamases (ESBLs), as well as plasmid-mediated AmpC enzymes, conferring resistance to many penicillins and third-generation cephalosporins.^11,12^ Chromosomal AmpC enzymes are inducible in response to certain β-lactam antibiotics and are most commonly encountered among *Enterobacter cloacae* complex, *Klebsiella aerogenes* and *Citrobacter freundii*.^12^ Carbapenem resistance is largely mediated by serine carbapenemases and MBLs.^13^ New Delhi MBL prevalence among Enterobacterales is increasing in the USA and has become the predominant carbapenemase in New York City.^7^

*P. aeruginosa* resistance relies on intrinsic mechanisms, such as efflux pump upregulation, porin loss and overexpression of chromosomal AmpC, augmented by acquired plasmid-mediated β-lactamases.^14^ Similarly, *A. baumannii* exhibits multilayered resistance via aminoglycoside-modifying enzymes, β-lactamases, porin changes and target-site mutations. Carbapenem resistance is primarily driven by oxacillinase-type carbapenemases, although MBLs are emerging.^15,16^

Multiple resistance mechanisms frequently coexist within a single isolate. For instance, β-lactamase production may occur alongside porin loss, efflux pump upregulation, or target-site mutations and may be a major driver of XDR phenotypes. Horizontal gene transfer (HGT) refers to bacterial acquisition of new resistance traits by acquiring genetic material from unrelated organisms.^17^ In Gram-negative bacteria, this occurs mainly through conjugation, in which plasmids carrying resistance genes are transferred between bacteria by cell-to-cell contact.^17,18^ Plasmids and other mobile genetic elements, such as integrons and transposons, facilitate capture, rearrangement and expression of resistance genes that cause resistance to multiple antibiotic classes.^9,17–19^ Transformation, involving uptake of free DNA, and transduction, in which bacteriophages transfer DNA between bacterial cells, also contribute to the spread of AMR.^17^ HGT introduces resistance genes into bacterial populations, while antibiotic selection ensures that bacteria carrying these genes have a survival advantage, allowing them to persist.^16,20,21^

While HGT facilitates the spread of resistance genes, clonal expansion refers to the transmission of a single bacterial strain that is already resistant.^21,22^ Clonal spread can lead to hospital-acquired outbreaks of antibiotic-resistant Gram-negative bacteria.^23^

## Transmission dynamics

**Figure 1.**
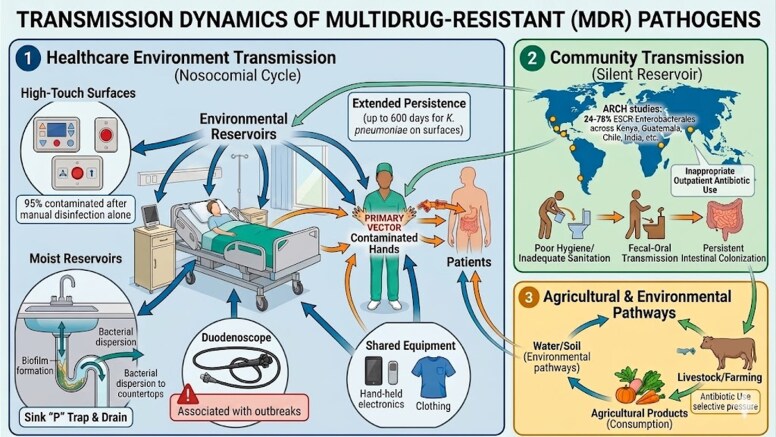
Transmission dynamics of multidrug-resistant pathogens. Abbreviations: ARCH, Antibiotic Resistance in Communities and Hospitals; ESCR, extended-spectrum cephalosporin-resistant Enterobacterales.

A combination of genetic, ecological and healthcare-related factors facilitates the emergence and transmission of resistant bacteria (Figure 1). Transmission in healthcare settings is mediated by reservoirs, such as contaminated environmental surfaces, water sources (especially the ‘P’ trap of sinks), patients and shared medical equipment. From any reservoir, contaminated hands are the primary vector for pathogen transmission to new patients.^24^ Clinically important nosocomial pathogens—including *E. coli*, *Acinetobacter* spp. and *Klebsiella pneumoniae*—can survive on surfaces in healthcare environments for extended periods, with the longest reported survival being 600 days for *K. pneumoniae*.^25^ Survival times varied by surface type, with porous surfaces associated with longer persistence.^25^

Contaminated medical equipment, clothing, cloth surfaces and hand-held electronics can facilitate ongoing dissemination within patient care areas.^26^ In a 2016 study evaluating the use of hydrogen peroxide vapour disinfection systems to reduce patient exposure to hospital pathogens, 214 sites were tested, and 95% remained contaminated with bacteria after manual disinfection alone. High contamination levels were identified on frequently touched surfaces, such as bed control panels and nurse call buttons.^27^ Furthermore, endoscopes (especially duodenoscopes) are associated with outbreaks involving MDR organisms, and while current guidelines recommend high-level disinfection after each use to mitigate transmission risk, some experts have suggested sterilization of gastrointestinal endoscopes between patients.^28–30^

Moist environments, such as sinks and drains, support bacterial persistence through biofilm formation.^31^ Bacterial dispersion from a sink strainer to a countertop has been observed in an experimental setting.^32^ Furthermore, a clinical study identified the presence of carbapenemase-producing bacteria in 24% of sinks assessed at a tertiary academic medical centre.^33^ In 127 cases, a concordant carbapenemase was identified in the culture from a swab of the sink and a sample from the patient in the room. The study could not always determine whether the sink or patient had been colonized first.

Community transmission remains an important mechanism for the spread of MDR-GNB. Inadequate sanitation, poor hygiene practices and inappropriate antibiotic use in outpatient settings promote faecal–oral transmission and persistent intestinal colonization.^19,22,34^ Additionally, antibiotic use in agriculture and animal care exerts selective pressure that favours resistant bacteria, which can be transmitted through consumption of agricultural or animal meat products or by environmental pathways.^19,20^ Antibiotic use in agriculture or animal care has a direct effect on the recipient of the antibiotic, but it also has further effects through the presence of resistant bacteria and genes in the excrement of animals and of humans who consume these plants or animals. Furthermore, waste from antimicrobial manufacturing contains levels of antibiotics that exceed selective concentrations as well as relatively high levels of resistant bacteria and antibiotic resistance genes.^19^ Wastewater from healthcare settings themselves contains resistant bacteria.^20^ These dynamics contribute to proliferation of AMR GNB in the community. The Antibiotic Resistance in Communities and Hospitals studies reported substantial prevalence of extended-spectrum cephalosporin-resistant Enterobacterales in community settings, ranging from 24% to 78% across Kenya, Guatemala, Chile, India, Botswana and Bangladesh, highlighting a significant reservoir of resistance beyond healthcare facilities.^35^

## Hand hygiene

Hand hygiene (HH) is essential for reducing the transmission of MDR-GNB in healthcare settings. Consistent HH during patient care significantly decreases acquisition and colonization with MDR-GNB, particularly when part of a comprehensive, multimodal approach to infection prevention.^36^ As such, guidelines from the Society for Healthcare Epidemiology of America (SHEA) recommend specific quantities and locations of alcohol-based hand sanitizer dispensers depending on room privacy and workflow.^37^ Importantly, with rare exceptions, MDR-GNB do not exhibit reduced susceptibility to antiseptics (e.g. ethanol).

Despite the centrality of HH to infection prevention across all settings, assessment of and long-term compliance with recommended HH practices are challenging globally.^38,39^ High workload and workflow barriers hinder consistent performance.^39,40^ Surfaces serve as reservoirs for MDR-GNB; therefore, environmental contamination contributes to hand contamination, even when HH compliance is high.^39,40^

Assessing HH compliance is inherently complex. Direct observation is susceptible to sampling bias and the Hawthorne effect.^41^ Electronic monitoring systems face barriers related to high cost, concerns about their accuracy and workflow integration.^42^ In spite of these challenges, integrated HH programmes, supported by committed leadership, with transparent feedback to staff, remain crucial for mitigating MDR-GNB transmission.

## Contact precautions

The use of contact precautions (CP) for MDR-GNB remains foundational to infection prevention in acute care settings. CP refers to a bundle of measures that are implemented in addition to standard precautions to prevent the transmission of pathogens that spread via contact. In acute care settings, CP includes the isolation of patients and the use of gowns and gloves by healthcare workers.^43^ Some institutions include the use of dedicated disposable patient care equipment, such as stethoscopes.^44^ There is also considerable variability in initiation, duration and the specific organisms for which CP is enacted.^45,46^ Universal CP strategies may be appropriate during outbreaks, in high-acuity units or in settings with uncontrolled transmission of high-risk MDR-GNB, but can also strain healthcare resources, increase personal protective equipment (PPE) utilization and waste and contribute to adverse patient outcomes.^26,47,48^ As MDR-GNB become increasingly endemic, universal CP approaches are being re-evaluated in favour of more targeted, risk-based strategies.

The de-implementation of CP for ESBL-producing Enterobacterales (ESBL-E) represents a notable shift in infection prevention practice. Multiple institutions have discontinued CP for ESBL-E without increases in healthcare-associated transmission while also conferring substantial reductions in isolation-related costs.^49,50^ Selective de-implementation may allow infection prevention resources to be redirected towards organisms with greater epidemic potential while also reducing isolation-associated patient harms.^50^

In contrast, CPs remain standard in acute care hospitals for non-ESBL MDR-GNB, primarily CRE, CRAB and carbapenem-resistant *P. aeruginosa* (CRPA).^51,52^ Implementation has increasingly shifted from phenotype-based precautions towards approaches incorporating resistance mechanisms, anticipated duration of colonization and care setting.^53^ Phenotypic carbapenem resistance identified by antimicrobial susceptibility testing may overestimate transmission risk, as resistance can arise through chromosomal mechanisms, such as porin loss combined with ESBL or AmpC production.^9^ In contrast, carbapenemase-producing organisms are associated with plasmid-mediated resistance, outbreaks and interspecies spread, thus prompting prioritization of CP for confirmed or suspected carbapenemase producers.

ESCMID guidelines recommend single-room isolation of colonized or infected patients to reduce acquisition of MDR-GNB.^54^ In settings where single-room isolation is limited, cohorting is a pragmatic alternative.^55^ Cohorting may prioritize shared resistance mechanisms (e.g. same carbapenemase) over bacterial species,^56^ while mixing patients with distinct resistance mechanisms may increase the risk of cross-transmission and HGT.^56,57^ This approach to isolation and cohorting decisions is limited by access to molecular testing.^58^

Determining the appropriate duration of CP for MDR-GNB remains challenging due to prolonged and intermittent colonization, limited sensitivity of surveillance cultures and the absence of validated clearance criteria.^59,60^ Expert guidance from SHEA supports organism- and mechanism-specific approaches rather than fixed durations.^61^ For carbapenemase-producing CRE, existing guidelines commonly recommend prolonged or indefinite CP, although several institutions have reported successful discontinuation of CP after multiple negative surveillance cultures.^52,62^ Similar practices for CRPA have been reported, with some facilities reassessing CP after defined periods without positive cultures, particularly outside high-risk units.^51^ In contrast, CP approaches to CRAB are typically more conservative, given its environmental persistence and association with healthcare-associated outbreaks, with CP often maintained indefinitely without routine attempts at microbiologic clearance.^51,52^ Overall, the lack of standardized clearance protocols may lead to substantial institutional variability, with more restrictive approaches reserved for organisms with higher transmission risk. As the epidemiology of AMR evolves and new data emerge, CP strategies will require ongoing reassessment.

Adherence to the appropriate use of PPE can be monitored for patients who meet the institutional indications for CPs, with feedback for improvement provided to staff. Studies have reported a wide range of adherence to elements of CP bundles, 44%–100%.^44,63,64^ Monitoring of CP bundle adherence is recommended by ESCMID and SHEA, although evidence showing association with decreased incidence of MDR-GNB is limited.^43,54,65^

## Cleaning and disinfection

As previously mentioned, high-touch surfaces (e.g. bed rails, IV poles, patient over-bed tables, call buttons, and door handles) are reservoirs for MDR-GNB pathogens; however, studies show that high-touch surfaces are not necessarily more contaminated, possibly due to cleaning practices, but that surfaces close to the contaminant exhibit higher contamination.^66^ Although existing practice recommendations prioritize cleaning and disinfection of high-touch surfaces, an evidence-based argument could be made for focusing on all touchable surfaces.^65,67,68^ Shared medical equipment can also transmit pathogens. One randomized trial showed that dedicating personnel to low-level disinfection of shared medical equipment reduced healthcare-associated infection (HAI) prevalence in the intervention arm compared with control (9.8% versus 14.2%, approximately 130 fewer infections over a 36-week period).^69^

Cleanliness of surfaces in rooms can be measured after room cleaning to provide feedback to environmental services personnel.^70^ Swab of a specified surface area can be sent for culture to measure bacterial burden via aerobic colony count, although the result requires time to grow organisms.^71^ Adenosine triphosphate bioluminescence from a surface swab or fluorescent markers on a surface can measure bioburden rather than the presence of organisms *per se*, but provide real-time feedback at the time of measurement.^72–75^

Various disinfectants demonstrate efficacy against MDR-GNB, including quaternary ammonium compounds, peracetic acid and chlorine dioxide-based disinfectants.^76–79^ Other disinfection methods include UV-C light devices and hydrogen peroxide systems for terminal disinfection of patient rooms, which demonstrate some benefit in reducing bioburden; however, these methods have not been proven to eradicate MDR-GNB.^80,81^

Sterilization and high-level disinfection of medical equipment can successfully eliminate MDR-GNB organisms, but failure to perform this adequately has been linked to transmission.^82^ Adequate cleaning must always precede sterilization and high-level disinfection. The 2025 SHEA multisociety sterilization and high-level disinfection guidelines recommend sterilization and/or high-level disinfection techniques based on infection risk (critical, semi-critical and non-critical) of the medical devices in question.^82^ Complex, lumened devices pose unique challenges in high-level disinfection due to biofilm formation and inability to visualize internal device components; utilization of positive pressure techniques, high-resolution video inspection and adherence to device manufacturer instructions for use can ensure adequate cleaning.^82^

Intentional design of the healthcare environment is crucial to promoting infection control. Challenges with eradication of MDR-GNB have led to an interest in sinkless, and even waterless, ICUs. A recent systematic review suggested that waterless interventions (e.g. sinkless patient care areas, waterless bath products, and bottled water for consumption) helped terminate hospital-acquired MDR-GNB outbreaks.^83^ A separate systematic review identified studies suggesting removal of sinks from patient rooms or the use of water filters on taps reduced the risk of GNB acquisition but called for prospective trials to better evaluate interventions on sinks.^84^

## Screening/surveillance

Disease due to MDR-GNB may originate from asymptomatic intestinal colonization. Systematic reviews estimate the risk of progression to disease among CRE-colonized patients to range from 11% to 16.5%.^85,86^ Accordingly, identifying asymptomatic colonization via screening is increasingly important for the control of MDR-GNB.^87^

Surveillance of MDR-GNB can be active or passive; passive surveillance relies on cultures obtained during routine care, whereas active surveillance involves directed collection of specimens from asymptomatic patients and provides a more accurate estimate of colonization prevalence. One single-centre study found that only 7% of multidrug-resistant organism (MDRO) carriers were identified via passive surveillance.^88^ Additionally, active surveillance measures the baseline incidence of MDR-GNB in the target population and identifies when a significant increase should trigger an outbreak investigation.

Active surveillance can be targeted to high-risk wards or patients.^85^ The European Centre for Disease Prevention and Control recommends risk-based active surveillance at the time of hospital admission; high-risk attributes include ‘history of an overnight stay in a healthcare setting in the last 12 months, dialysis-dependent or cancer chemotherapy in the last 12 months, known carriage of CRE in the last 12 months and epidemiological linkage to a known carrier of a CRE’.^89^ High-risk patients undergo pre-emptive contact isolation and active screening for CRE.^89^

Active surveillance has also been implemented for CRAB. A multi-phase single-centre prospective study of hospital-acquired CRAB demonstrated that a multifaceted infection control programme did not decrease the incidence of HA-CRAB until large-scale screening was implemented.^90^

Screening for intestinal colonization by MDR-GNB is performed by analysing faecal samples (stool, perianal/perirectal or rectal swabs) via culture or molecular-based methods.^87^ Rapid molecular diagnostic methods include biochemical assays for carbapenemase detection, PCR-based identification of resistance genes (CTX-M, KPC, NDM) or multiplexed PCR and microarray panels.^9^ Culture is less expensive than molecular methods and can identify a wider range of resistant organisms by phenotype. Culture is preferred when there are many resistance mechanisms, as seen with ESBLs. Molecular-based methods have a shorter turn-around time and can specify the resistance mechanism. Molecular methods are effective when a clinical assay can target the relevant resistance mechanisms, as seen with carbapenemases in Enterobacterales.

## Decolonization

Decolonization could liberate patients from isolation precautions or reduce the risk of subsequent disease.^59^ An important caveat is that the criteria used for decolonization impact results—multiple negative perianal swabs provide greater specificity for decolonization than just one.^59,87^ A meta-analysis estimated that, in the absence of intervention, 55% of patients remained colonized with MDR Enterobacterales 6 months after initial detection.^59^

Studies have not identified an optimal intervention to hasten decolonization. Selective digestive decontamination (SDD) uses oral non-absorbable antibiotics and a short course of intravenous antibiotics to reduce the burden of enteric organisms and may prevent systemic disease. Studies of SDD have shown decreased colonization with MDR bacteria, bloodstream infections and ventilator-associated pneumonias.^91–93^ However, studies have heterogeneity in antibiotic choice and dose and target population. A recent large international randomized clinical trial failed to show a mortality benefit for SDD, although it showed a decrease in cultures with antibiotic-resistant organisms.^94^ The optimal clinical population for SDD has not yet been defined.

Daily chlorhexidine (CHG) treatment (i.e. bathing) has been shown to reduce bloodstream and MRSA infections in ICU patients.^95,96^ In skilled nursing facilities and acute care, CHG treatment has reduced positive cultures with MDR Enterobacterales.^97,98^ CHG treatment for patients in ICUs or oncology units, with central lines or in skilled nursing facilities currently has the strongest evidence base among interventions to reduce facility burden of MDR Gram-negative pathogens.

Faecal microbiota-altering interventions have theoretical potential to aid MDR Enterobacterales decolonization, and one study showed shortened time to decolonization of patients with carbapenemase-producing bacteria. However, faecal microbiota-altering interventions currently lack large prospective studies showing benefit.^87^

We recommend that patients identified to be colonized or infected with MDR-GNB should receive daily CHG treatment if they are in a high-risk inpatient unit or a skilled nursing facility or have a central line.^95–98^ SDD, faecal microbiota-altering interventions or other decolonization strategies do not have sufficient evidence to make a recommendation. Determining the most effective decolonization strategies and target populations is an important goal for future study.

## Antimicrobial and diagnostic stewardship

Optimizing diagnosis and treatment through antibiotic and diagnostic stewardship reduces the healthcare reservoir of pathogens and the likelihood of transmission. Therefore, stewardship programmes are essential to mitigating outbreaks and selection pressure contributing to resistance. Structural frameworks for stewardship, such as CDC’s Core Elements of Hospital Antibiotic Stewardship programmes, recommend multiple evidence-based actions, such as prescription audit and feedback, formulary restriction to help steer antibiotic selection, pre-authorization of broad-spectrum antibiotics and education on judicious and appropriate antibiotic use. These frameworks also recommend evidence-based interventions in collaboration with hospital partners, such as microbiology, infection prevention and control (IPC) programmes and information technology specialists, to develop and implement systems for optimal execution of antibiotic stewardship. In particular, close collaboration with the microbiology laboratory ensures (i) timely diagnosis and effective treatment of individual infections and (ii) implementation of proven methods to optimize antibiotic selection across the acute care population. These methods include facility or unit-wide antibiograms, selective reporting and cascading of susceptibility results. While antibiograms provide evidence-based guidance for empiric antibiotic selection, selective reporting and cascades steer optimal prescribing by emphasizing the use of the narrowest effective therapies and encouraging stepwise escalation only when narrower agents are resistant.

Microbiology and stewardship collaboration is also essential for the effective implementation of rapid molecular diagnostics to identify resistance genes and expedite treatment. Carba-R or BioFire^®^ Film^®^ Blood Culture Identification Panel 2 can hasten time to contact isolation for patients with extensively drug-resistant bacteria. Results can inform targeted antibiotic therapy as per Table 1.^10,99^ Rapid diagnostics support the care continuum of identification, rapid isolation and treatment for the acute care patient population.

Likewise, effective stewardship–IPC collaboration ensures avoidance of over-testing, diagnosis and treatment of common hospital conditions, such as *Clostridioides difficile*, urinary tract infection, blood culture contamination, etc.

## Special settings

Patients in special settings with a higher risk of acquiring infections may benefit from tailored interventions.

A systematic review found that a four-component strategy was most effective for preventing acquisition of MDR-GNB in the ICU setting, including standard and transmission-based precautions, antimicrobial stewardship programmes, environmental cleaning and CHG bathing.^100^ The risk of MDR-GNB infection from devices in the ICU or other settings can be reduced by the same interventions that reduce the risk from bacteria in general, as described in SHEA practice recommendations for central line associated blood stream infections, ventilator associated pneumonia and catheter associated urinary tract infections.^101–103^

MDR-GNB colonization persists longer in immunocompromised patients, leading to an elevated risk of MDR-GNB infections due to extended hospitalizations, immunosuppressing medications, the necessity of indwelling devices and exposure to broad-spectrum antibiotics.^104^ Due to prolonged carriage, surveillance of up to 3 years for ESBL-producing *Klebsiella*, OXA-48–producing organisms and MDR *Pseudomonas* has been suggested.^60^ Haematopoietic stem cell transplant recipients should preferably be placed in private rooms with positive pressure and HEPA filtration.^105^ Positive pressure ventilation primarily protects against airborne fungal pathogens, whereas the private room helps reduce contact and environmental transmission of MDR-GNB.

## Resource-limited settings

IPC practices are critical for limiting HAIs and AMR in low- and middle-income countries (LMICs), where the burden of MDR-GNB is disproportionately high.^106^ This is driven by multiple factors, including suboptimal IPC practices and the widespread overuse of antimicrobials across human medicine, veterinary medicine and agriculture. Consequently, efforts to combat AMR in LMICs must address these underlying drivers while acknowledging resource restraints.

IPC in LMICs faces substantial challenges, particularly in high-risk settings, such as the ICU.^107^ Studies consistently show that HAIs are significantly more prevalent in LMICs than in high-income countries, driven by limited IPC infrastructure, financial and human resources, overcrowding, heavy workloads and inadequate training of healthcare workers. Poor compliance with basic IPC measures—especially HH—combined with insufficient access to personal protective equipment, clean water and diagnostic microbiology, facilitates cross-transmission and contributes to the high burden of MDROs. Late establishment of IPC programmes, lack of surveillance systems and inappropriate antibiotic use further exacerbate AMR and HAIs in LMICs.^108,109^

Despite these limitations, evidence suggests that implementing simple, cost-effective and context-appropriate IPC interventions—such as strengthening HH, establishing basic surveillance, forming dedicated IPC teams and applying care bundle approaches—can significantly reduce infection rates and improve patient outcomes in resource-limited settings.^110–113^

The WHO Infection Prevention and Control Assessment Framework (WHO IPCAF) provides a standardized, evidence-based approach to evaluate and strengthen IPC programmes at national and facility levels. In LMICs, IPCAF identifies gaps, prioritizes interventions and guides stepwise implementation of IPC measures according to available resources.^114^ By operationalizing the eight WHO IPC core components, IPCAF enables healthcare facilities to move beyond fragmented practices towards structured, sustainable IPC programmes, making it particularly relevant for controlling MDR-GNB.^115^

IPC strategies in resource-limited settings must therefore be pragmatic, context adapted and focused on high-impact interventions to reduce transmission of MDR-GNB. In alignment with IPCAF, establishment of functional IPC programmes at the facility level (Core Component 1) is foundational but frequently undermined by structural constraints, such as overcrowding; inadequate water, sanitation and hygiene infrastructure; limited laboratory capacity; and shortages of trained personnel. These limitations often restrict the implementation of IPC guidelines originally developed for high-income settings (Core Component 2).^114–116^

Consequently, simplified and prioritized IPC bundles targeting HH, environmental cleaning and basic surveillance are emphasized as core elements of multimodal IPC strategies (Core Component 5). Innovative, low-cost solutions, including local production of alcohol-based hand rub, task shifting to trained IPC champions and the use of visual reminders, strengthen IPC education and training (Core Component 3) and substantially improve compliance despite infrastructural limitations.^114,116^ For MDR-GNB, pragmatic measures, including patient cohorting, rational use of invasive devices, and early removal of invasive devices, address key risks related to workload, staffing, and bed occupancy (Core Component 7).^114,115^

Surveillance for HAIs and MDR-GNB (Core Component 4) remains challenging in many LMICs due to limited access to microbiology; nevertheless, syndromic surveillance, outbreak detection and targeted monitoring can guide IPC responses and antimicrobial stewardship. Continuous monitoring, audit and feedback (Core Component 6), delivered in a non-punitive manner, are essential to sustain adherence to IPC practices. Finally, improvements in the built environment, materials and equipment (Core Component 8), particularly environmental cleaning and reprocessing of shared medical equipment, are critical for controlling environmentally persistent pathogens, such as CRE and CRAB.^114,117,118^

Overall, the implementation of the WHO IPCAF framework provides a solid foundation for reducing the burden of MDR-GNB in healthcare settings in LMICs, by enabling consistent, scalable and sustainable infection control practices.

## Future directions

Future directions regarding the prevention and control of MDR-GNB within healthcare settings will be shaped by technological advances in automating surveillance, the use of large language models and predictive technologies for early identification, containment and treatment of MDR-GNB pathogens. Automated systems should identify and report pathogens earlier, enable faster intervention and reduce the time required by infection prevention personnel to conduct this work. Moreover, electronic health record advances have enabled better tracking of patients across various healthcare settings to minimize horizontal spread to vulnerable patient populations (i.e. long-term care settings and chronic ventilator facilities). Finally, artificial intelligence (AI) chatbots can also be used to generate educational information for providers, patients and families pertaining to care within the healthcare facility and upon return to the community. Incorporation of AI into IPC requires validation of the accuracy of data outputs, assurance of patient confidentiality and reduction of biases before widespread adoption.^119^

### Conclusion

In conclusion, infection prevention and control of MDR-GNB presents a unique challenge within the healthcare setting. Table 2 summarizes recommendations of the authors based on expert opinion and considerations discussed in this review article. While evidence-based strategies, such as HH, CPs and standardized cleaning and disinfection protocols, are the mainstay of IPC for MDR-GNB pathogens, the complexity lies in the evolving and multifaceted nature of drug resistance and effective treatment, which is an important aspect of prevention of horizontal spread. While rapid molecular diagnostics can more efficiently identify resistance mechanisms and help diagnose MDR-GNB infections, universal availability of diagnostics and effective treatments remain limited, especially in resource-constrained settings. Moreover, due to potentially refractory infections or prolonged colonization, certain host populations may require intensified or extended isolation strategies, and optimal duration of CPs for various pathogens, resistance mechanisms and special populations requires further study. However, decolonization strategies, such as CHG bathing and SDD, show promise as adjunctive IPC strategies for preventing the spread of MDR-GNB organisms. Finally, advances in surveillance technology within electronic health records may improve the efficiency of surveillance processes and the application of effective control methods.

**Table 2.** Recommendations to prevent transmission of MDR-GNB in healthcare settings

Scope	Recommendation
Regional/national	Stewardship of outpatient human antibiotics
	Stewardship of antibiotic use in agriculture and animal husbandry
Low- and middle-income countries	Implement the WHO Infection Prevention and Control Assessment Framework to identify highest yield strategies to reduce MDR-GNB infections
Healthcare facilities	Monitor adherence to institutional hand hygiene policy
	Use contact precautions for carbapenem-resistant or other high-risk MDR-GNB
	Monitor adherence to the proper use of PPE for contact precautions
	Monitor effectiveness of room cleaning
	Assure competency of staff who perform high-level disinfection and sterilization of medical devices
	Implement daily chlorhexidine bath treatment for ICU and high-risk oncology populations, patients with central venous catheters and patients in skilled nursing facilities
	Implement CDC core elements of hospital antibiotic stewardship
	Implement rapid diagnostic tests to support early identification of antimicrobial resistance with mechanism
Unresolved issues requiring further research	De-implementation of contact precautions for low-risk MDR-GNB
	Duration of contact precautions for MDR-GNB
	Removal of sinks from ICU or other patient rooms
	Effectiveness of active screening for MDR-GNB; optimal patient populations and target organisms for active screening
	Optimal decolonization protocol for MDR-GNB and whether this varies with organism

MDR-GNB, multidrug-resistant Gram-negative bacteria.
